# Histological validation of dynamic-equilibrium cardiovascular magnetic resonance for the measurement of myocardial extracellular volume

**DOI:** 10.1186/1532-429X-15-S1-O16

**Published:** 2013-01-30

**Authors:** Christopher A Miller, Josephine H Naish, Paul Bishop, Glyn Coutts, David Clark, Sha Zhao, Simon G Ray, Nizar Yonan, Simon G Williams, Andrew Flett, James Moon, Geoffrey Parker, Matthias Schmitt

**Affiliations:** 1 North West Heart Centre and Transplant Unit, University Hospital of South Manchester, Manchester, UK; 2 Biomedical Imaging Institute, University of Manchester, Manchester, UK; 3 Department of Pathology, University Hospital of South Manchester, Manchester, UK; 4 Alliance Cardiac MRI Unit, University Hospital of South Manchester, Manchester, UK; 5 Christie Medical Physics and Engineering, The Christie Hospital, Manchester, UK; 6 The Heart Hospital, London, UK; 7 Institute of Cardiovascular Science, University College London, London, UK

## Background

Extracellular matrix expansion is fundamental to left ventricular (LV) remodeling, and is a therapeutic target. CMR techniques are increasingly used to evaluate myocardial extracellular volume (ECV), however the most widely applied methods are without histological validation. The aim of this study was to provide whole-heart, histological validation of; 1. Dynamic-equilibrium CMR (DynEq-CMR), where ECV is quantified using hematocrit-adjusted myocardial and blood T1 values measured before and after gadolinium bolus; and 2. Isolated measurement of myocardial T1 at a fixed time-point following gadolinium bolus, used as an ECV surrogate.

## Methods

**Figure 1:**
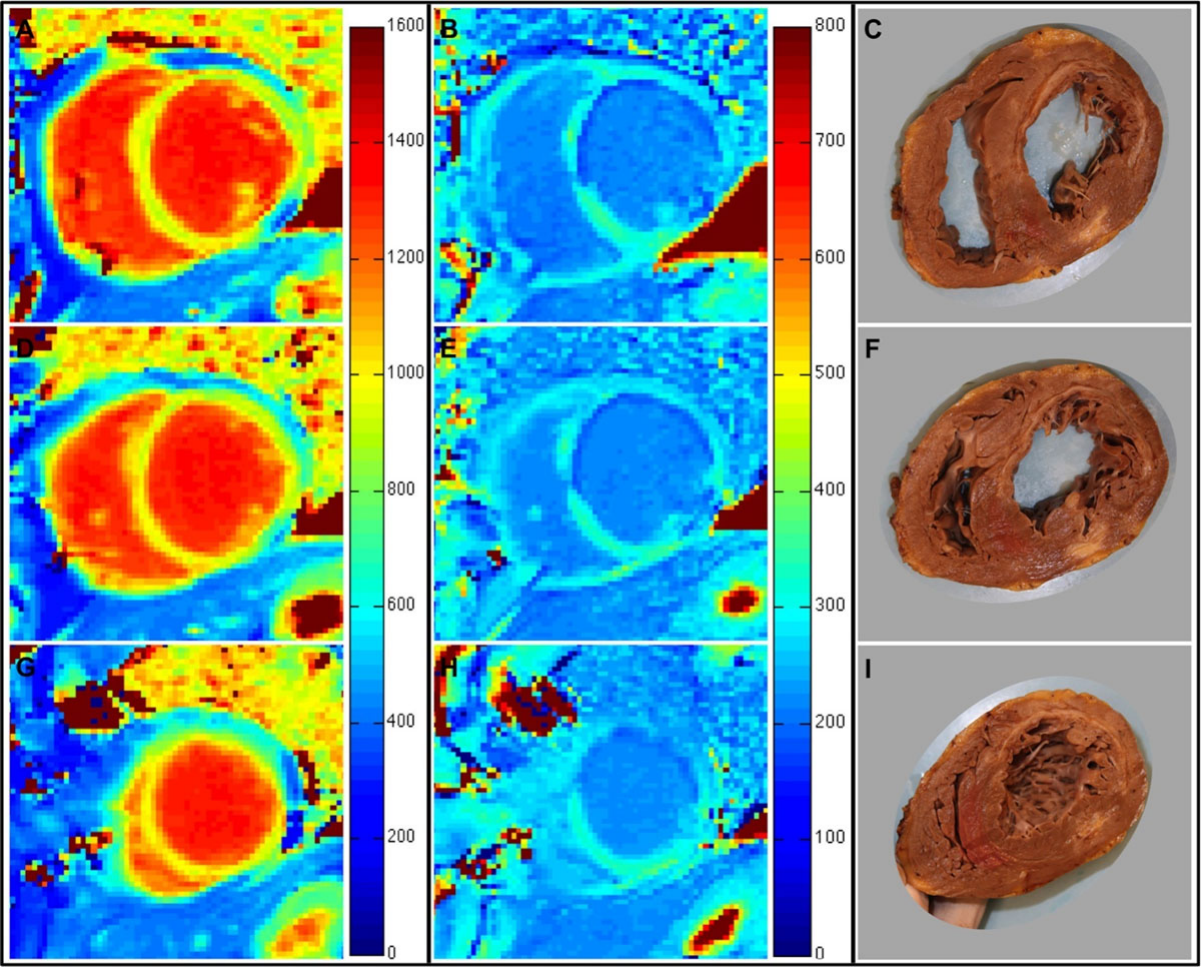
Histological validation of dynamic-equilibrium CMR. Short-axis pixelwise T1 relaxation time maps at basal (A and B; colors correspond to T1 relaxation time according to colour bar legends), mid (D and E) and apical (G and H) ventricular levels before (A, D, G) and 15-minutes after a bolus of 0.20mmol/Kg Gd-DTPA contrast (B, E, H), were determined from modified Look Locker inversion recovery imaging in order to determine segmental myocardial extracellular volume fraction. Subsequently, patients underwent heart transplantation. Explanted hearts were immediately fixed in 10% buffered formalin and cut at corresponding basal (C), mid (F) and apical (I) ventricular levels. Tissue blocks were taken from each segment before being embedded in paraffin and stained with picrosirius red in order to determine segmental histological collagen volume fraction.

CMR was performed prospectively in patients awaiting heart transplantation. Of 54 patients on the transplant waiting list at a single UK Center between Jan 1 2011 and July 1 2012, 41 had contraindications to CMR, 2 were too unwell and 2 refused consent. The remaining 9 underwent CMR, including modified look locker inversion recovery imaging at base, mid and apical LV levels before and 10 and 15mins after 0.2mmol/Kg Gd-DTPA bolus at 1.5T, and same-day hematocrit measurement. Resulting pixelwise T1 maps (MatLab) were used to calculate segmental ECV. (Phantom studies performed prior to patient scanning determined T1 measurement accuracy and heart-rate correction algorithm). 6 patients subsequently underwent transplantation (median interval between CMR and transplant 29 days). 16 tissue samples taken from each heart according to the 16-segment model (96 segments in total) were analysed for picrosirius red collagen volume fraction (CVF, Figure 1). The same CMR protocol was also performed in 10 matched healthy subjects.

## Results

**Figure 2:**
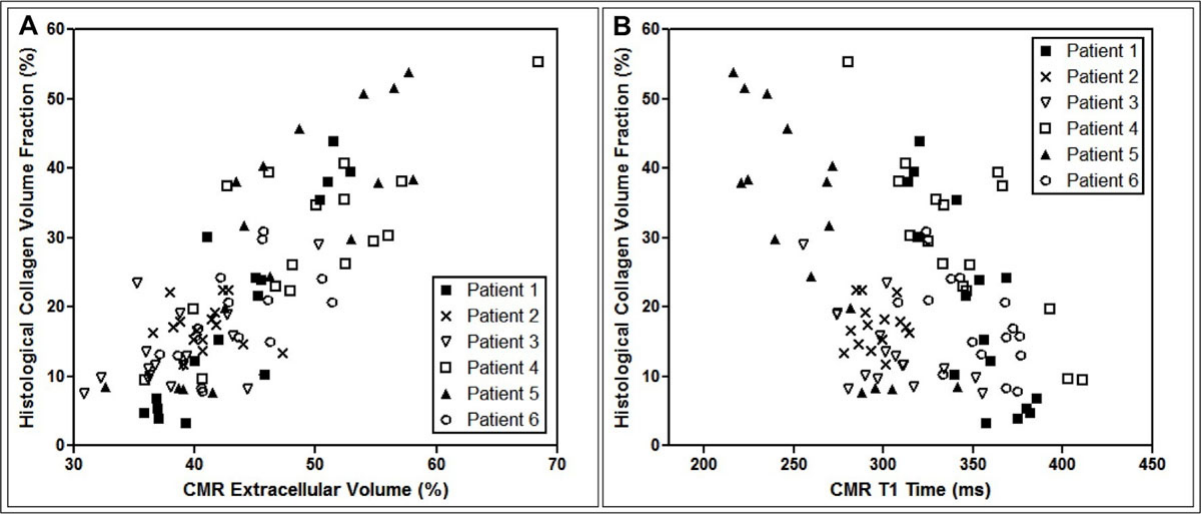
(A) Dynamic-equilibrium CMR-measured myocardial extracellular volume plotted against histological collagen volume fraction. (B) Isolated post-contrast T1 measurements made at 15-minutes post-contrast plotted against histological collagen volume fraction. Symbols correspond to different patients, as set out in the legends.

DynEq-CMR-derived ECV was linearly related to histological CVF (p<0.01; within-subject r=0.75, p<0.01; r2=0.56; between-subject r=0.95, p<0.01, r2=0.89; for ECV calculated using 15min post-contrast T1; linear regression equation: histological CVF=1.45xECV-42, Figure 2). Correlation was maintained throughout the entire heart (i.e. across all ventricular levels and septal and non-septal segments), and when segments displaying late gadolinium enhancement (LGE) were included and excluded. Isolated post-contrast T1 measurement showed significant within-subject correlation with histological CVF (r=-0.74, p<0.01; r2=0.55 for 15min post-contrast T1), but between-subject correlations were not significant, likely reflecting between subject confounding factors such as renal function and body habitus. Pre-contrast T1 values and histological CVF were not significantly correlated. Mean segmental ECV in segments without LGE (41.4±5.0%) and with LGE (47.0±7.4%) were both significantly higher than in healthy subjects (25.5±2.6%; p<0.01).

## Conclusions

DynEq-CMR-derived ECV shows a good correlation with histological CVF throughout the whole heart. Isolated post-contrast T1 measurement is insufficient for ECV assessment.

## Funding

Christopher Miller is supported by a Doctoral Research Fellowship from the National Institute of Health Research (UK).

